# The role of anti-core exercises in public health: a preventive approach to spinal health

**DOI:** 10.1590/1806-9282.20250667

**Published:** 2026-05-01

**Authors:** Meric Odemıs, Halil Orbay Cobanoglu, Hale Bozkurt Cobanoglu

**Affiliations:** 1 Alanya Alaaddin Keykubat University, Sport Sciences Faculty – Alanya, Türkiye.; 2 Alanya Alaaddin Keykubat University, Alanya Education and Research Hospital, Antalya Provincial Health Directorate, Department of Medical Microbiology – Alanya, Türkiye.

Dear Editor,

Low back pain (LBP) represents a leading cause of disability worldwide, contributing to decreased quality of life and increased economic burden on healthcare systems. The Lancet Commission reports that approximately 540 million people are affected globally, with rising prevalence linked to sedentary behavior, aging populations, and poor postural health^1^. While traditional core strengthening exercises (e.g., sit-ups, back extensions) are commonly integrated into rehabilitation programs, recent attention has turned to anti-core stabilization training—a group of exercises that enhance neuromuscular control by resisting movement rather than generating it.

Anti-core movements (e.g., anti-rotation, anti-extension, and anti-lateral flexion) activate deep spinal stabilizers and reduce shear forces on the spine without imposing excessive compressive loads, especially in vulnerable populations such as older adults or sedentary workers. A meta-analysis by Hlaing et al. reported significant reductions in pain and disability following core stabilization programs compared to traditional strengthening exercises^2^. Similarly, Smrcina et al. demonstrated that patients engaging in core stability interventions experienced greater improvements in trunk control and daily function than those following general exercise routines^3^.

Despite this growing evidence base, anti-core approaches remain underutilized in public health frameworks and are often absent in national guidelines or workplace wellness initiatives. Notably, the NICE guideline (NICE, 2020) recommends structured physical activity for chronic LBP but does not differentiate between core exercise types. A gap exists between clinical efficacy and community-level implementation^4^.

## Public health implementation framework

To address this gap, structured and scalable delivery methods must be developed. First, target populations should be clearly defined. The ideal candidates include sedentary office workers, older adults at risk of falls, postnatal women with trunk instability, and individuals recovering from musculoskeletal injuries.

Implementation models should emphasize simplicity, accessibility, and minimal equipment. Short anti-core routines (5–10 min), using bodyweight movements such as front planks, bird dogs, and dead bugs, can be integrated into:

- Workplace wellness programs during scheduled breaks,
- School physical education curriculums,
- Community health screenings,
- Geriatric fall prevention initiatives in primary care.

For instance, in a randomized controlled trial by Akhtar et al., a 6-week core stabilization program led to a significantly greater reduction in pain (Visual Analog Scale [VAS] score improvement: 3.08±1.49) compared to routine exercise therapy (VAS improvement: 1.71±1.52) in patients with chronic non-specific low back pain^5^.

Barriers to implementation include exercise literacy, inconsistent supervision, perceived lack of time, and access to instructional material or equipment (e.g., resistance bands for Pallof presses). To overcome these, public health authorities should promote mobile applications, video tutorials, and community-based instruction tailored to various literacy levels and cultures.

While the long-term cost-effectiveness of such programs remains under-investigated, the low-risk profile and broad applicability of anti-core exercises justify their inclusion in preventive care efforts. Future public health research should include pilot studies evaluating adherence, functional outcomes, and the health economic impact of anti-core protocols in real-world settings.

Anti-core stabilization exercises offer biomechanical efficiency, neuromuscular activation, and low-risk profiles. Their implementation in public health policy can fill a critical gap between clinical success and preventive practice. We call on researchers, clinicians, and policymakers to consider these exercises not only as an athletic adjunct but also as an accessible strategy to combat the rising global burden of LBP.

## Data Availability

The datasets generated and/or analyzed during the current study are available from the corresponding author upon reasonable request.
